# Phylogenetic relationship of some “accessory” helicases of plant positive-stranded RNA viruses: toward understanding the evolution of triple gene block

**DOI:** 10.3389/fmicb.2015.00508

**Published:** 2015-05-19

**Authors:** Sergey Y. Morozov, Andrey G. Solovyev

**Affiliations:** A. N. Belozersky Institute of Physico-Chemical Biology, Moscow State UniversityMoscow, Russia

**Keywords:** plant virus, virus genome, RNA helicase, evolution, movement protein, triple gene block

## Abstract

Recently, we hypothesized that silencing suppression activity gained by a viral replicative helicase led to the emergence of the second helicase possessing activity of the viral silencing suppressor and/or movement protein (MP). Our hypothesis accounted for the evolutionary origin of the specialized ‘triple gene block’ (TGB) in plant virus genomes encoding the MPs TGB1, TGB2, and TGB3 required for viral cell-to-cell transport through plasmodesmata. Here, we used public transcriptome databases to identify previously unrecognized viruses. The analysis of novel viral genomes further supported the previously proposed scenario of TGB origin and evolution, which included the following steps. First, the accessory helicase gene could have been acquired by horizontal gene transfer (HGT) presumably occured independently in different virus groups. Second, the TGB2 gene evolved by HGT or autonomization of the C-terminal transmembrane domain found in at least one TGB1 helicase. Third, the TGB3 gene has most likely emerged in the genomic block consisting of the TGB1 and TGB2 genes.

## Introduction

For replicative RNA/DNA synthesis, plant viruses encode enzymes belonging to one of three helicase superfamilies (SFs), SF-I, SF-II, and SF-III (Koonin and Dolja, 1993; Iyer et al., 2004; Hickman and Dyda, 2005; Jankowsky and Fairman, 2007; Byrd and Raney, 2012; Gilhooly et al., 2013). Participation of replicative helicases of all three SFs in virus cell-to-cell movement and silencing suppression is documented (Wang et al., 2012, 2014; Sorel et al., 2014). Particularly, the replicative SF-I RNA helicase domain of distantly related tobamoviruses may play an important role in the suppression of silencing (Kubota et al., 2003; Ding et al., 2004; Csorba et al., 2007; Wang et al., 2012). Recently, assuming the participation of plant virus helicases in non-replicative functions, we proposed a hypothetic scheme, which explains evolving the triple gene block (TGB) in plant viruses (Morozov and Solovyev, 2012). TGB is a module consisting of three genes termed TGB1, TGB2, and TGB3, which encode movement proteins (MPs) necessary for viral cell-to-cell transport. TGB2 and TGB3 encode integral membrane proteins, whereas the TGB1 protein contains a helicase domain, which belongs to a diverged lineage of viral SF-I helicases (Morozov and Solovyev, 2003; Verchot-Lubicz et al., 2010; Solovyev et al., 2012). In agreement with the earlier view that the TGB1 protein can be regarded as an “accessory” helicase evolved after duplication of a replicative RNA helicase (Koonin and Dolja, 1993), we hypothesized that gaining new silencing suppression function by a replicative RNA helicase could precede subsequent helicase domain duplication concomitant with its autonomization, which can occur both *in cis* (in the context of the same viral genome) or *in trans* (implying transfer to a foreign virus genome). These events may result in evolving specialized second helicase possessing the activity of viral silencing suppressor (VSR), or, taking into account a tight link between viral movement and silencing suppression, both suppression and movement functions (Burgyán and Havelda, 2011; Morozov and Solovyev, 2012; Pumplin and Voinnet, 2013). Examples of such helicases are provided by potexvirus and carlavirus TGB1 proteins having the VSR function additional to their cell-to-cell movement function (Bayne et al., 1995; Senshu et al., 2011). Further evolution could be accompanied by loss of VSR function by the replicative helicase. We assumed that further TGB1 specialization as a dedicated MP could be accompanied by acquisition of TGB2 and TGB3 genes facilitating the TGB1-mediated cell-to-cell transport (Morozov and Solovyev, 2012).

Based on sequence comparisons and functional studies, two types of TGB were distinguished, a ‘potex-like’ TGB encoded by filamentous viruses of the families *Alphaflexiviridae* and *Betaflexiviridae* and a ‘hordei-like TGB’ found in rigid rod-shaped viruses of the family *Virgaviridae* and the unassigned genus *Benyvirus*. Apart from these well-characterized TGB-containing viruses, the second “accessory” helicase gene was identified in genomes of a number of diverse unclassified plant viruses of different genome organization and particle morphology, particularly, *Nicotiana velutina mosaic virus* (NVMV) and *Hibiscus green spot virus* (HGSV; Morozov and Solovyev, 2003, 2012; Verchot-Lubicz et al., 2010).

In recent years, the identification of previously unknown viral genomes has been greatly accelerated with high-throughput sequencing technologies. New-generation sequencing of plant transcriptomes often gives rise, in addition to species-specific libraries of mRNA sequences assembled from many individual reads (Johnson et al., 2012; Xie et al., 2014), to virus-like RNA assemblies (VLRA) corresponding to genomic RNAs of viruses infecting these host plants. Information obtained from VLRA, full-length or even partial, can be used to discover new viral proteins and domains. As a result, numerous novel genotypes of eukaryotic viruses were identified in last several years (Rosario and Breitbart, 2011; Cook et al., 2013; Junglen and Drosten, 2013; Roossinck, 2015). In this paper, in an attempt to further resolve potential evolutionary relations between distant TGB-containing viruses, we performed new comparative sequence analyses of “accessory” helicases encoded in previously sequenced plant virus genomes, including NVMV and HGSV, and VLRAs found in public databases, particularly, the 1000 Plants (1KP) project (Matasci et al., 2014).

## Nicotiana Velutina Mosaic Virus

**FIGURE 1:**
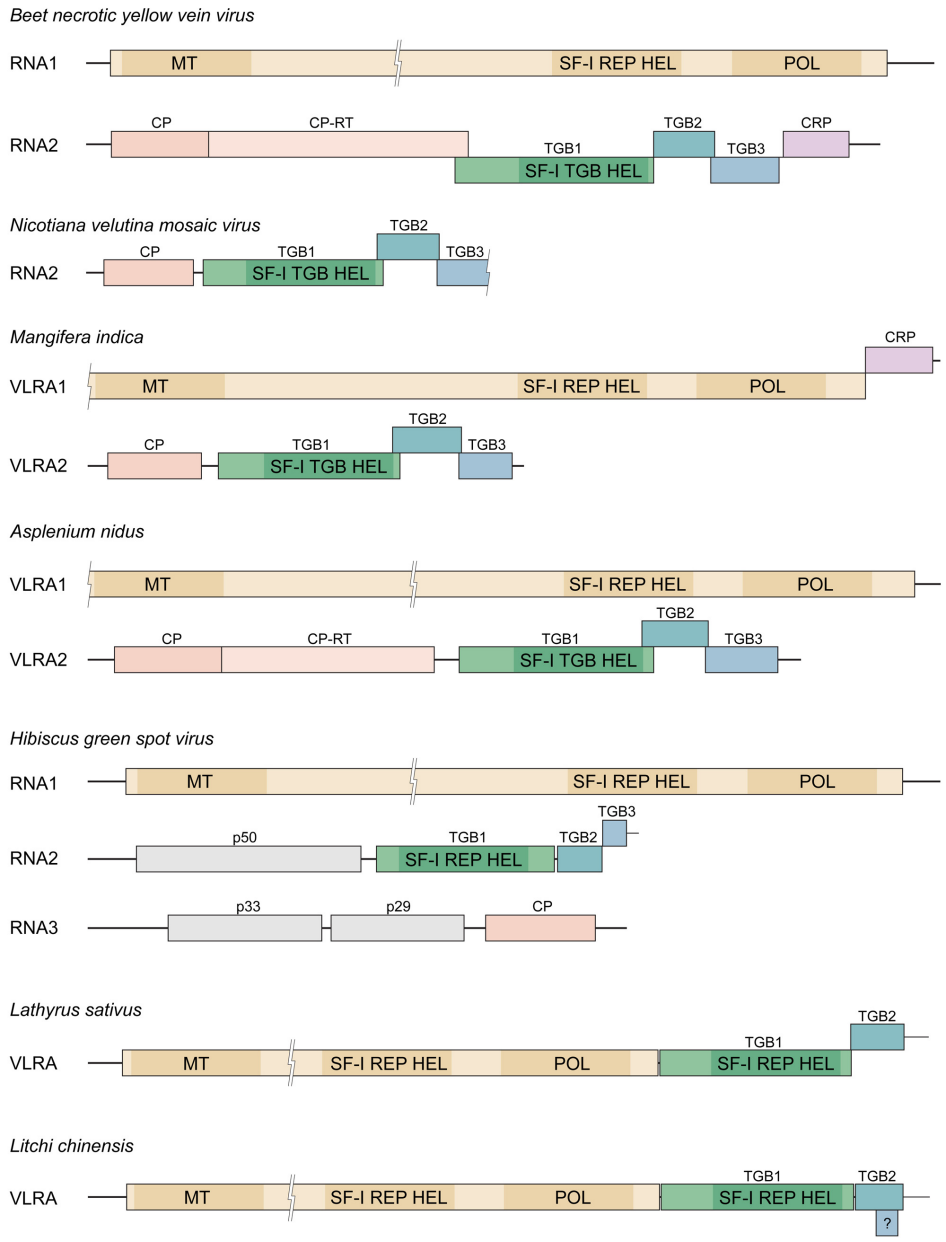
**Triple gene block (TGB) in genomes of different plant viruses and virus-like RNA assemblies (VLRAs)**. Genes are shown as boxes, and molecular masses of the encoded proteins with unknown function are indicated. TGB is shown in green. Genes of replicative proteins are shown in yellow. The locations of conserved protein sequence domains of methyltransferase (MT), helicase (HEL) and polymerase (POL) are indicated by shading. Other domains in replicative proteins are not shown. “SF-I REP HEL” denotes the superfamily I helicase domain typical for replicase proteins, “SF-I TGB HEL” – the superfamily I helicase domain typical for TGB1 proteins. CP, coat protein gene; CP-RT, readthrough domain of CPs; CRP, genes of cysteine-rich proteins. “?” indicates the short open reading frame (ORF) in Lc-VLRA potentially representing TGB3 gene.

*Nicotiana velutina mosaic virus* genome consists of RNA1 (8 Kb) and RNA2 (3 Kb), which are encapsidated in rigid, rod-shaped particles (Randles and Rohde, 1990). NVMV RNA1 sequence is unavailable, while incomplete sequence of RNA2 revealed four open reading frames (ORFs; (**Figure 1**). The 5′-terminal ORF1 encodes a protein showing relatively weak but significant similarity to coat proteins (CPs) of viruses of the genus *Benyvirus* such as *Beet necrotic yellow vein virus* (BNYVV), whereas the ORF2 protein was also found to have closest relationship to benyvirus TGB1 “accessory” helicases (identity 28–30%; Randles and Rohde, 1990). NVMV ORF3 protein represents typical TGB2 protein with two terminal hydrophobic regions and highly conserved hydrophilic signature in the central part. Although the NVMV ORF4 was sequenced only partly, the encoded protein was found to have benyvirus-like TGB3 organization with two hydrophobic regions at N- and C-termini (Randles and Rohde, 1990; Morozov and Solovyev, 2003). However, NVMV TGB3 had only marginal (if any) sequence relationship to benyvirus TGB3 proteins in its central hydrophilic region. It should be noted that this region is variable even in known benyvirus TGB3 sequences (Kondo et al., 2013).

TBLASTN search of NCBI transcriptome shotgun assembly (TSA) database with NVMV CP and ORF2 protein as queries revealed their significant similarity to proteins encoded by a single VLRA (size 2523 nucleotides) of mango (*Mangifera indica*; **Table 1**). This mango VLRA (referred to as Mi-VLRA2) contains four ORFs (**Figure 1**); ORF1 and ORF2 code for proteins similar to NVMV CP and TGB1, respectively, whereas ORF3-encoded protein can be aligned with the NVMV TGB2 protein (identity 47%; **Table 1**). The mango VLRA2 ORF4 protein has the typical TGB3 organization with the N- and C-terminal hydrophobic segments (Solovyev et al., 2012), but only marginal sequence similarity to the NVMV TGB3 protein that is not surprising in view of low sequence conservation between benyvirus and NVMV TGB3 proteins. Thus Mi-VLRA2 is similar to NVMV RNA2 both in genetic organization and the encoded proteins.

**Table 1:** Comparisons of amino acid sequence identities of the proteins encoded by Triple gene block (TGB) containing virus genomes and virus-like RNA assemblies (VLRAs).

Query open reading frames (ORF)	Subject ORF	E-value	Maximal amino acid identity (%)	Nucleotide accession numbers
	***Mangifera indica* Mi-VLRA1**			NCBI GBCV01010547
Replication protein^∗∗^	Rice stripe necrosis benyvirus Replication protein	3e-171	61	NCBI EU099844
	Burdock mottle benyvirus Replication protein	3e-167	58	NCBI AB818898
	Beet necrotic yellow vein benyvirus Replication protein	3e-166	57	NCBI EU330453
	Beet soil-borne mosaic benyvirus Replication protein	8e-165	56	NCBI JF513082
	***Mangifera indica* Mi VLRA2**			NCBI GBCV01004715
Coat protein (CP)	Nicotiana velutina mosaic virus (NVMV) CP	7e-26	40	NCBI D00906
	Rice stripe necrosis benyvirus CP	2e-07	30	NCBI EU099845
	Beet necrotic yellow vein benyvirus CP	5e-04	26	NCBI AY771345
	Beet virus Qpomovirus CP	0.003	25	NCBI EU785970
TGB1	NVMV TGB1	3e-42	33	NCBI D00906
	Burdock mottle benyvirus TGB1	4e-28	33	NCBI AB818899
	Rice stripe necrosis benyvirus TGB1	1e-27	31	NCBI EU099845
	Beet necrotic yellow vein benyvirus TGB1	3e-23	28	NCBI X75575
	Beet soil-borne mosaic benyvirus TGB1	3e-23	27	NCBI JF513083
TGB2^∗^	NVMV TGB2	3e-13	52	NCBI D00906
	Potato mop-top pomoviru TGB2	1e-06	34	NCBI JX885628
	Broad bean necrosis pomovirus TGB2	1e-04	32	NCBI D86638
	***Asplenium nidus* An-VLRA1**			1KP database PSKY-2058769
Replication protein^∗∗^	Rice stripe necrosis benyvirus Replication protein	2e-153	34	NCBI EU099844
	Burdock mottle benyvirus Replication protein	5e-143	33	NCBI AB818898
	Beet necrotic yellow vein benyvirus Replication protein	5e-139	31	NCBI EU330453
	Beet soil-borne mosaic benyvirus Replication protein	3e-136	31	NCBI JF513082
	***A. nidus* An-VLRA2**			1KP database PSKY-2058768
CP	Peanut clump pecluvirus CP	3.3	27	NCBI NP620028
	Ribgrass mosaic virus CP	2.1	31	NCBI CAJ13878
	Tobacco mosaic virus CP	3.2	26	NCBI AAA46587
TGB1	Burdock mottle benyvirus TGB1	1e-29	35	NCBI AB818899
	Rice stripe necrosis benyvirus TGB1	8e-29	31	NCBI EU099845
	Beet soil-borne mosaic benyvirus TGB1	4e-24	31	NCBI JF513083
	Beet necrotic yellow vein benyvirus TGB1	3e-23	31	NCBI X75575
	NVMV TGB1	2e-22	29	NCBI D00906


TGB2^∗^	Beet necrotic yellow vein benyvirus TGB2	5e-10	41	NCBI AY682694
	Rice stripe necrosis benyvirus TGB2	5e-10	44	NCBI EU099845
	Burdock mottle benyvirus TGB2	1e-07	36	NCBI AB818899
	***Litchi chinensis* Lc-VLRA**			1KP database WAXR-2010981
TGB1	Hibiscus green spot virus (HGSV) TGB1	4e-36	37	NCBI HQ852053
	Burdock mottle benyvirus Replication HEL	1e-13	32	NCBI AB818898
	Rice stripe necrosis benyvirus Replication HEL	3e-11	28	NCBI EU099844
	Beet soil-borne mosaic benyvirus Replication HEL	1e-10	29	NCBI JF513082
	Beet necrotic yellow vein benyvirus Replication HEL	2e-09	28	NCBI EU330453
	Ferret hepatitis E virus Replication HEL	2e-08	25	NCBI AB890001
Replication protein^∗∗∗^	Beet soil-borne mosaic benyvirus Replication protein	5e-110	32	NCBI JF513082
	Burdock mottle benyvirus Replication protein	5e-70	31	NCBI AB818898
	***Lathyrus sativus* Ls-VLRA**			NCBI GBSS01016353
TGB1	HGSV TGB1	2e-37	34	NCBI HQ852053
	Rice stripe necrosis benyvirus Replication HEL	2e-10	29	NCBI EU099844
	Beet necrotic yellow vein benyvirus Replication HEL	8e-10	27	NCBI EU330453
	Beet soil-borne mosaic benyvirus Replication HEL	4e-09	27	NCBI JF513082
Replication protein^∗∗∗^	Burdock mottle benyvirus Replication protein	9e-87	33	NCBI AB818898
	Beet necrotic yellow vein benyvirus Replication protein	6e-72	34	NCBI EU330453

*^∗^more than 60% query coverage*.

*^∗∗^POL domain only*.

*^∗∗∗^MET, HEL, and POL domains*.

Interestingly, NVMV TGB1 was additionally found to have a distant similarity to a protein encoded by another mango VLRA1 (size 4934 nucleotides). Mi-VLRA1 contains two ORFs (**Figure 1**). The 5′-terminal large ORF includes methyl/guanylyltransferase (MET), cysteine protease (PRO), SF-I helicase (HEL), and RNA polymerase (POL) domains similar to those in the replication protein of benyviruses (**Table 1**), whereas the 3′-terminal ORF show distant relationship to benyvirus cysteine-rich proteins (data not shown). We speculate that Mi-VLRA1 and Mi-VLRA2 may represent a new genome type of TGB-possessing viruses having larger and smaller genome components organized similarly to pecluvirus RNA1 and hordeivirus RNAβ, respectively, (Morozov and Solovyev, 2003). In the absence of NVMV RNA1 sequence, possible relation of this putative mango virus to NVMV remains to be investigated. We found no similarity of nucleotide sequences at the 5′ and 3′ termini between longer and shorter mango VLRAs that may be a result of incomplete sequencing of terminal regions.

TBLASTN search of 1KP database (www.onekp.com), which contains around 1000 plant transcriptomes, with NVMV ORF2 protein as a query showed significant similarity to a single VLRA (size 4138 nucleotides) of fern *Asplenium nidus* (**Table 1**). This fern VLRA (referred to as An-VLRA2) contains five ORFs similar to benyvirus RNA2 genes in both sequences of encoded proteins and linear arrangement excepting the lack of 3′-terminal CRP gene in An-VLRA2 (**Figure 1**; Kondo et al., 2013). The encoded proteins are related also to those of beny-, pomo-, and pecluviruses (**Table 1**). Strikingly, the putative TGB3 protein encoded by fern An-VLRA2, despite the characteristic benyvirus-type TGB3 organization with two terminal hydrophobic segments (Solovyev et al., 2012), showed a weak similarity to the central hydrophilic region of TGB2 proteins encoded by potex- and carlaviruses rather than to any known TGB3 protein (data not shown). This observation reinforces our previous conclusion on possible diverse evolutionary origins of TGB3 sequences in distant viruses (Morozov and Solovyev, 2003).

*Nicotiana velutina mosaic virus* TGB1 protein was also found to have a distant similarity to a protein encoded by another *A. nidus* An-VLRA1 (size 6960 nucleotides). The fern An-VLRA1 contains a single ORF (**Figure 1**), which is obviously similar to the replication protein of benyviruses in its HEL, PRO, and POL domains (**Table 1**). We speculate that the two fern VLRAs represent a new viral genome. As in the case of mango VLRAs, we found no similarity of nucleotide sequences at the 5′ and 3′ termini between longer and shorter fern VLRAs.

## Hibiscus Green Spot Virus

*Hibiscus green spot virus* (proposed genus *Higrevirus*) genome consists of three RNAs which are encapsidated in short, bacilliform particles of 30–50 nm. These RNAs are designated as RNA1 (8.35 Kb), RNA2 (3.17 Kb), and RNA3 (3.11 Kb; Melzer et al., 2012). HGSV RNA1 has a single ORF encoding the replicase protein, which contains MET, PRO, SF-I helicase (HEL), and POL domains (**Figure 1**) and shows most significant similarity to replicases of plant cileviruses, furoviruses, and pomoviruses as well as insect negeviruses (Melzer et al., 2012; Vasilakis et al., 2013). RNA2 possesses four ORFs including three TGB genes, whereas RNA3 contains at least three ORFs (**Figure 1**; Melzer et al., 2012). The HGSV TGB1 helicase is very distantly related to other TGB1 proteins and shows more similarity to the SF-I replicative helicases of the genus *Benyvirus* (**Figure 1**). HGSV TGB2 is distantly related to other TGB2 proteins; short HGSV TGB3 (47 aa in length) contains two long hydrophobic segments with extremely small central hydrophilic region and shows no similarity to any other TGB3 protein (Solovyev et al., 2012).

TBLASTN searches of NCBI and 1KP databases with HGSV TGB1 protein as a query revealed significant similarity (higher than to any viral sequences) to two long VLRAs of plants *Lathyrus sativus* (7970 nucleotides) and *Litchi chinensis* (7388 nucleotides; **Table 1**; **Figure 1**). HGSV TGB2 protein was also best aligned with the proteins encoded by these Lc-VLRA and Ls-VLRA (data not shown). Despite sequence similarity to HGSV (TGB proteins) and benyviruses (HEL–POL domains), it should be noted that VLRAs of *L. sativus* and *L. chinensis* represent another type of genome organization, which is more similar to TGB-containing viruses of the families *Alphaflexiviridae* and *Betaflexiviridae* (Morozov and Solovyev, 2003). However, the most remarkable features of the Ls-VLRA and Lc-VLRA are (1) the sequence relation of the TGB2 signatures in the central hydrophilic region (conserved also in HGSV TGB2) to the TGB3 of As-VLRA2 (data not shown) and (2) the absence of TGB3-like ORFs downstream of TGB2 genes. Interestingly, Ls-VLRA has a small ORF, which is located within the TGB2 gene in a different reading frame, encoding a small (42 aa in length) hydrophobic TGB3-like protein (**Figure 1**). These findings strongly support our previous suggestion (Morozov and Solovyev, 2012) that the second step of the TGB module formation in plant virus evolution could be the acquisition of TGB2-like protein in addition to an autonomized helicase domain. Existence of this stage in the TGB evolution, previously merely hypothetical, is strongly supported by finding Ls-VLRA, which encodes no TGB3 protein. On the other hand, Lc-VLRA TGB3-like ORF might illustrate the TGB3 gene origination by overprinting of TGB2.

## Virus Genomes Encoding Both SF-I and SF-II Helicases

An initial step of the TGB origination could take place as duplication or horizontal gene transfer (HGT) of the replicative helicase domain (Morozov and Solovyev, 2012). If we consider the acquisition of an additional helicase gene *per se* (not in the context of TGB evolution), available examples of viral genomes with non-TGB “accessory” helicases suggest that this process could occur through HGT. The genome of *Chara australis virus* (CAV; Gibbs et al., 2011) encodes a large protein, which shows the relationship with RNA polymerases of benyviruses, and the CP related to the CP of tobamoviruses. The helicase domain of this replicative protein belongs to SF-I. Two additional CAV ORFs code for a non-replicative RNA helicase and a protein of unknown function. Importantly, this CAV helicase is related to CI helicase (SF-II) of Ipomoviruses (family *Potyviridae*). RNA virus with a similar genome organization was recently found in charcoal rot (NCBI accession NC_025674). This fungal virus (*Macrophomina phaseolina tobamo-like virus*) encodes a large ORF with replicative MET/SF-I HEL/POL domains related to tobamoviruses and a SF-II helicase ORF showing highest sequence similarity to CI helicase of *Potato virus Y* (family *Potyviridae*; data not shown).

One additional example of HGT resulting in a combination of SF-I and SF-II helicases in single virus genome is provided by endornavirus *Gremmeniella abietina type B RNA virus XL* (GaBRV-XL; Tuomivirta et al., 2009). Members of the genus *Endornavirus* (the family *Endornaviridae*) have linear double-stranded RNA genomes of 9.8–17.6 kb in length containing one ORF coding for a single polypeptide, which is thought to be processed by a proteinase. The encoded polyprotein comprises conserved POL domain and, in most cases, SF-I RNA helicase domain (Song et al., 2013). The second SF-II DExH box helicase of GaBRV-XL is most similar to the HEL domain from *Classical swine fever virus* (the family *Flaviviridae*, genus *Pestivirus*; Tuomivirta et al., 2009). Endornaviruses seem not to form true virions and found in plants, fungi, and protists (Song et al., 2013). Emergence of a second SF-II helicase in addition to unrelated replicative SF-I helicase in the CAV, Macrophomina virus, and GaBRV-XL genomes is intriguing assuming that no definite function can be attributed so far to these accessory helicases.

## Posible Scenarios of TGB Evolution

The transition to multicellularity could be beneficial for life forms, which had been earlier evolved to acquire mechanisms of antiviral silencing at a single-cell evolutionary stage. Indeed, small RNA, rapidly moving between the cells of a primordial multicellular organism ahead of the advancing infection, could increase the immunity of distant cell and, consequently, the viability of the multicellular organism as it was described for present-day plants (Hyun et al., 2011; Melnyk et al., 2011; Amari et al., 2012). Along with PCD (Iranzo et al., 2014), this defense mechanism could provide evolutionary advantages to multicellular organisms over communities of unicellular organisms, serving therefore as a driving force in the evolution of multicellularity. We have speculated that plus-RNA viruses of unicellular algae in the course of transition of their hosts to multicellularity may have evolved additional RNA helicase genes by shuﬄing with distantly related viruses or by duplication of helicase domain in their own replicase. These novel genes combined VSR and MP functions for efficient spread over the multicellular plant organism (Morozov and Solovyev, 2012). Further evolution of virus genomes could have resulted in the origination of the separated MP and VSR proteins.

Considering the possible mechanisms of evolving the genes coding for such proteins, one can propose a combination of horizontal transfer (HGT) and *de novo* origin by duplication and/or overprinting (Rancurel et al., 2009; Pavesi et al., 2013). Particularly, the MPs of *Alphaflexiviridae* and *Betaflexiviridae* are located in the same genomic position (downstream of the replicase gene) but encoded by either the TGB type or the single MP gene (Martelli et al., 2007). Somewhat different situation was found in *Tombusviridae* and *Virgaviridae,* where the transport module is positioned differently among virus genera and consists of the single MP gene or double gene block/TGB, respectively, (Morozov and Solovyev, 2003; Stuart et al., 2004; Adams et al., 2009; Schoelz et al., 2011). These facts strongly indicate that at least one type of MPs have been acquired by viruses of these families by HGT. HGT may be also considered as the most probable evolutionary event in acquisition of SF-II helicase gene by plant virus CAV, fungal Macrophomina virus, or GaBRV-XL endornavirus.

New observations reported here allowed us to extend the understanding of possible scenarios for TGB evolution and its successive steps. First, accessory helicase gene was acquired in the process of HGT or gene duplication that might occur independently in different virus groups. Second, the TGB2 gene could have been evolved by HGT or autonomization of the C-terminal transmembrane domain, found in at least one TGB1 helicase, by a frame-shift mutation bringing the future TGB2 sequence into another reading frame (Morozov and Solovyev, 2012).

The TGB3 gene has most likely evolved in the genomic block consisting of TGB1 and TGB2 genes at the latest step of TGB formation. The TGB3 gene, which often overlaps the TGB2 gene, was predicted to appear by overprinting, in which an existing coding sequence is becoming to be translated in two reading frames (Rancurel et al., 2009; Morozov and Solovyev, 2012). A possible loss of virus fitness due to its reduced ability to tolerate mutations in gene overlap regions can be compensated by an evolutionary advantage, as it was found in studies of the paramyxovirus overlapping P/C genes coding for identical functional motifs (Lo et al., 2014). In the case of TGB2 and TGB3 it may be advantageous to encode essential functional hydrophobic motifs in overlapping frames (provided that they are rather short). Finally, an important feature of overlapping genes is that they provide a regulatory advantage that may recompense the increased constraints they impose on the virus, by encoding two proteins that are co-regulated and have coordinated functions (Pavesi et al., 2013). Indeed, the expression of the TGB2 and TGB3 proteins is co-regulated, since they are transcribed from the same messenger RNA (Verchot-Lubicz et al., 2010; Solovyev et al., 2012).

In the evolution of different TGB types, the TGB3 gene could originate by the process of overprinting, the TGB2 gene duplication and subsequent divergence of the emerged TGB3 gene, or an acquisition of an TGB2-independent sequence encoding a progenitor of the TGB3 gene. Possible independent origin of small TGB3 genes in some virus families explains the structural and functional diversity of TGB3 proteins identified among viruses sequenced so far (Morozov and Solovyev, 2012). Impressive example of this evolutionary diversity could present HGSV-related TGBs. Indeed, HGSV TGB3 gene is encoded just downstream TGB2 gene; potential TGB3 ORF in Lc-VLRA is located completely within the TGB2 gene (**Figure 1**), and Ls-VLRA possesses no identifiable TGB3 ORF. Therefore, the presented analysis of new virus and virus-like sequences confirms the previously suggested scenario for the TGB evolution and provides new models as well as a framework for further functional studies of non-canonical TGB modules.

In general, analysis of plant VLRAs appeared to be a powerful tool, which can help to shed a new light on the details of diversity and evolution of RNA viruses, and, particularly, TGB-containing genomes. Additionally, new TGB-possessing VLRAs can provide new experimental models for exploration of TGB protein functions. Deep sequencing technologies have enabled detection of both known and novel viruses with unprecedented sensitivity. However, the large numbers of reads generated by these methods necessitate new approaches for filtering raw data and discriminating putative viral transcripts for further detailed analysis. In should be mentioned that, in addition to TGB helicases, we used viral RNA-dependent RNA polymerase signatures to select plant virus sequence homologs in the TSA sequence data. The identified transcripts carrying potential viral signatures have enabled the reconstruction of dozens of previously unknown, nearly complete viral genomes from overlapping reads (to be published elsewhere). Therefore, we further expand our analysis to plant TSA libraries in a search for unknown ssRNA viruses.

## Conflict of Interest Statement

The authors declare that the research was conducted in the absence of any commercial or financial relationships that could be construed as a potential conflict of interest.
